# Prevalence of onchocerciasis and associated clinical manifestations in selected hypoendemic communities in Ghana following long-term administration of ivermectin

**DOI:** 10.1186/s12879-019-4076-2

**Published:** 2019-05-17

**Authors:** Kenneth Bentum Otabil, Samuel Fosu Gyasi, Esi Awuah, Daniels Obeng-Ofori, Robert Junior Atta-Nyarko, Dominic Andoh, Beatrice Conduah, Lawrence Agbenyikey, Philip Aseidu, Comfort Blessing Ankrah, Abdul Razak Nuhu, H. D. F. H. Schallig

**Affiliations:** 1 Department of Basic and Applied Biology, School of Science, University of Energy and Natural Resources, Sunyani, Ghana; 2 Amsterdam University Medical Centres, Academic Medical Centre, University of Amsterdam, Department of Medical Microbiology, Experimental Parasitology Unit, Amsterdam, The Netherlands; 3 Department of Civil Engineering, Kwame Nkrumah University of Science and Technology, Kumasi, Ghana; 4 Office of the Vice Chancellor, Catholic University College of Ghana, Sunyani, Ghana; 5 Department of Community Medicine and Health, Anglican University College of Technology, Nkoranza, Ghana

**Keywords:** Onchocerciasis, Nodules, Dermatitis, Hypoendemic areas, Neglected tropical disease, Ocular onchocerciasis

## Abstract

**Background:**

Onchocerciasis is a neglected tropical disease which is still of immense major public health concern in several areas of Africa and the Americas. The disease manifests either as ocular or as dermal onchocerciasis with several symptoms including itching, nodules, skin thickening, visual impairment and blindness. Ivermectin has been an efficient microfilaricide against the causative agent of the disease (*Onchocerca volvulus*) but reports from some areas in Africa suggest the development of resistance to this drug. The aim of this study was to determine the prevalence of onchocerciasis and associated clinical conditions frequently associated with the disease in three endemic communities in Ghana which have been subjected to 18 to 20 rounds of mass drug administration of ivermectin. This was to help determine whether or not onchocerciasis persists in these communities.

**Methods:**

A cross-sectional study design was adopted. Three communities (Tanfiano, Senya and Kokompe) in the Nkoranza North District of Ghana where mass drug administration of ivermectin had been ongoing for more than two decades were selected for the study. The population was randomly sampled and 114 participants recruited for the study based on the eligibility criteria. The study participants were examined for the presence of parasites and clinical manifestations of onchocerciasis following established protocols.

**Results:**

The study showed that the prevalence of microfilaria in the Tanfiano, Senya, Kokompe communities were 13.2, 2.4, and 2.9%, with nodule prevalence being 5.3, 4.9 and 14.3% respectively. Females in the study communities had a higher prevalence of microfilaria carriers (5.17%) relative to males (2.44%), but this difference was not statistically significant (*p = 0.2800, unpaired t test*). The most frequent clinical manifestation observed in this study among all participants was dermatitis (25.4%), followed by visual impairment & nodules (7.9% each) and then by blindness (4.4%).

**Conclusion:**

The study showed that despite several years of mass drug administration with ivermectin, infection with onchocerciasis and the commonly associated clinical manifestations of the disease still persist in the study communities. This calls for a greater urgency for research and development aimed at discovering new or repurposed anti-filarial agents which will augment ivermectin if global onchocerciasis eradication targets are to be achieved.

## Background

Onchocerciasis is the world’s second largest cause of infectious blindness and a major public health problem in many parts of the world [1]. The disease is caused by *Onchocerca volvulus* parasites and endemic in 37 countries in West, East and Central Africa, the Arabian Peninsula and parts of South and Central America [1]. Worldwide, about 90 million people are at risk of contracting the disease in endemic areas mainly in sub Saharan Africa, of which more than 37 million are estimated to be infected and 300,000 are permanently blind as a consequence of onchocerciasis [1] . However, these figures are generally accepted to be an underestimation of the true situation [2].

Although rarely life threatening, onchocerciasis causes chronic suffering and severe disability with approximately 1.5 million disability-adjusted life-years (DALYs) being lost each year due to this disease [3] . Severe pruritis associated with the disease alone accounts for 60% of these DALYs [3]. In some hyper-endemic communities, every second person infected ultimately goes blind [4]. Moreover, infection with *Onchocerca* worms reduces the host’s immunity and resistance to other diseases, resulting in an estimated reduction in life expectancy of 13 years [3]. If many people are infected in a community, socioeconomic depression may result as fertile riverine areas are frequently abandoned for fear of the disease [1].

Clinical features of the disease include both skin and ocular involvement. Typically, skin involvement consists of intense itching, swelling, and inflammation [1]. Skin changes include acute papular onchodermatitis, chronic papular onchodermatitis, hyperpigmentation, lichenified onchodermatitis, lymphadenopathy, pruritus and common secondary bacterial infections; Skin atrophy; depigmentation, usually on anterior lower leg [5]. Ocular involvement provides the common name associated with onchocerciasis - river blindness and may involve any part of the eye from conjunctiva and cornea to uvea and posterior segment including retina and optic nerve [5].

Currently, the only drug available to treat onchocerciasis is ivermectin (Mectizan TM, Merck) [6]. Ivermectin is a safe and effective microfilaricide and has since 1987 been successfully used in mass drug administration programs (MDA) aimed at both reducing the burden of disease and controlling transmission [6]. However, persistent microfilaridermia (microfilariae in the skin) and transmission have been reported after 15–20 years of ivermectin treatment in some areas of Africa [7–9]. Awadzi et al. (2004) working in Ghana identified *O. volvulus* worms that exhibited an ivermectin response phenotype termed sub-optimal response (SOR) [7, 10]. These SOR parasites were characterized by the presence of live stretched microfilariae in the uteri of the adult worms 90 days after treatment, and were associated with repopulation of the skin with microfilariae earlier and more extensively than expected based on prior data [10, 11]. Nevertheless, ivermectin exposure was found not to be an explanatory factor [10, 11]. Additional investigations have reported this phenotype in other areas in Ghana [2, 9, 12] and in Cameroon [13, 14]. Allele frequency change in a number of candidate ivermectin response genes (chosen for analysis based on specific hypotheses concerning mechanisms of resistance to the acute effects of ivermectin in *O. volvulus*) has also been demonstrated in these populations when sampled before and after several rounds of ivermectin treatment [8, 15–19], which is consistent with population genetic changes associated with drug selection pressure [20]. While genetic selection for SOR was not demonstrated, these studies suggest that these populations are being influenced at the genetic level by ivermectin treatment and if these candidate genes mediate the phenotypic changes in ivermectin response then SOR might have a genetic basis that may involve selection on several genes [20].

In response to the increasing concerns on diminishing effectiveness of ivermectin, there is a need to keep monitoring the efficacy of ivermectin in onchocerciasis endemic areas. The present study aimed to investigate the prevalence of onchocerciasis and clinical manifestations commonly associated with the disease in three communities in the middle belt of Ghana that are endemic for onchocerciasis and have been subjected to 18 to 20 rounds of MDA. The study aimed to provide information on the persistence (or otherwise) of the disease and its clinical manifestations in the study communities.

## Methods

### Study area

This study was done in 3 communities (Tanfiano, Senya and Kokompe) in the Nkoranza North District of the Bono East Region of Ghana (West Africa). The three communities were selected because no effective monitoring of the effectiveness of the MDA programmes in the communities had previously been performed in them, although ivermectin MDA had been ongoing in these endemic communities for several years. The communities were chosen to represent the Western (Tanfiano), central (kokrompe) and Eastern (Senya) communities of the District [21, 22]. The Nkoranza North lies within longitudes 1° 10″W and 1 ° 55″W and latitudes 7° 20″N and 7 ° 55″N [21, 22]. The Tanfi and Tanko Rivers in the area are fast flowing and provide suitable breeding grounds for the black flies which are the vectors of onchocerciasis. The major rainy season is between April and June and the minor rainy season occurs between September and November [21]. The temperature in the District is generally high with an annual average temperature of 26.0 °C. Average maximum temperature is 30.9 °C and minimum of 21.2 °C. The hottest months are February, March and April [21].

### Study design

A cross-sectional study with a random sampling approach was employed. The study was part of a larger study which started in September 2017 and ended in February 2019. The cross-sectional (prevalence) study was performed in September 2017. A few days prior to the recruitment, announcements were made via the community information centers or by a ‘gong-gong’ beater (a local means of giving information to community residents where the ‘beater’ moves around sounding a metallic instrument ‘the gong gong’ whilst intermittently shouting out the information). Community residents were informed to gather at chosen social centers. The study was explained in English and then in the local language ‘Twi’ during the meeting with the village elders and interested villagers at a chosen gathering point in the community. The participants were invited to ask questions and these were answered. During the surveys, persons identified as eligible to participate in the study were informed individually in English by the research team and then in ‘Twi’. Informed, signed/thumb printed, or witnessed consent was obtained from all participants. The Informed Consent Forms were in English and were explained in local language to participants who did could not read or write in English.

### Recruitment of study patients

A total of 114 eligible individuals from all 3 communities were randomly sampled and recruited for this study, resulting in an average sample size of 38 individuals from each community. This meets the average sample size (around 40 per village) for most prevalence studies on onchocerciasis as reported by the WHO [23]. Individuals were made to randomly queue and all individuals who were in the queue were given the chance of being included in the study once they met the inclusion criteria. The criteria for inclusion in the study was that a participant should be a male or female, aged 18–95 years with willingness to participate in the study, indicated by signing an informed consent form. The informed consent form along with study protocol was approved by the scientific and ethical review Boards of the Kintampo Health Research Centre (KHRC) in Ghana. The approval number for the study is KHRCIEC/2018–18. Before parasitological and physical examinations of eligible participants, basic demographic data, i.e. name, gender, age and number of rounds of ivermectin were collected using the case report forms.

### Palpation

Individuals were palpated to check for the presence of nodules (onchocercomas) harbouring adult worms of *O. volvulus*. Nodules were ‘suspected’ onchocercomas (not confirmed by dissection or biopsy) defined as being firm, often flattened or bean-shaped, usually movable, non-tender and up to several centimeters in diameter. They were distant from usual locations of lymph nodes (neck, axillae, inguinal) as the palpation were done on bony prominences of the ribs, iliac crests, sacrum and upper leg [24].

### Visual acuity tests

All study participants were examined by medical personnel for possible vision loss suspected to be associated with past or ongoing infection with onchocerciasis using a Snellen chart to determine whether the volunteers had 20/20 vision or if they showed signs of visual impairment [25]. Visual impairment was defined as presenting with visual acuity worse than 6/12 (mild), 6/18 (moderate) or 6/60 (severe) whereas blindness was defined as presenting visual acuity worse than 3/60. All examinations were done without corrective glasses.

### Physical examinations for signs of dermal onchocerciasis

The study participants were further examined for the signs of dermal onchocerciasis and the results of these assessments were recorded in the case reports forms [24]. Dermal onchocerciasis was defined as any of the common skin manifestations of onchocerciasis. An example is dermatitis which is the first manifestation and maybe isolated or associated with skin lesions [26]. It can be troublesome and sometimes irresistible though scratching may lead to filarial scabies which is often confused with scabies, especially in countries where suspicion of onchocerciasis does not arise [26].

### Skin snipping and microscopy

Tiny pieces of blood-free skin snips were obtained from all registered study participants from each community to determine individual microfilarial counts in order to evaluate the prevalence and intensity of infection in each community. A piece of cotton wool soaked in methylated spirit was used to clean both sides of the iliac-crest of each person. Blood-free skin snips (approx. 2 mg.) were taken from each individual using a sterilized corneoscleral punch. The biopsies were then incubated in physiological saline for microscopic examination to detect microfilariae (mf) of *O. volvulus* [2].

Microfilariae were counted using 10X objective lens of a compound microscope. The geometric means of the microfilaria from the 2 skin biopsies from each patient were calculated and used as a measure of intensity of infection. The standard prevalence was also determined as the cumulative prevalence of all persons infected in the population.

### Statistical analysis

Data analyses were done using Graph Pad Prism statistical software© (Version 5). The microfilaria prevalence was expressed as a percentage (number of persons positive for microfilaria divided by number examined × 100). Chi-square analysis, ANOVA and Welch’s unequal variances *t test* were used to test for differences in microfilaria and nodule prevalence within and between the study communities. A two-tailed *p-value* lower than 0.05 was considered statistically significant.

## Results

### Demographic data of study communities

#### Gender

In total 114 participants were included in the study from all the three study communities, of which 50.88% (*n* = 58) were females and 49.12% were males (*n* = 56).

#### Age distribution of study participants

The age distributions of study participants in all three study communities are presented in Table 1. In the Senya community, the study showed that the age group 51–61 years had the highest frequency of 14 representing about 34.15% of the total population sampled in the community (Table 1). The lowest frequency of participants (4) was recorded in the 29–39 years category.

**Table 1:** Frequency Distribution of Ages of Study Participants in Senya, Kokompe and Tanfiano Communities

Age Group	Frequency (percent)			
	Senya	Kokompe	Tanfiano	Total
18–28	8(19.5%)	1(2.9%)	7(18.4%)	16(14.0%)
29–39	4(9.8%)	4(11.4%)	8(21.1%)	16(14.0%)
40–50	9(22.0)	11(31.4%)	8(21.1%)	28(24.6%)
51–61	14(34.2%)	6(17.1%)	7(18.4%)	27(23.7%)
62–95	6(14.6%)	13(37.1%)	8(21.1%)	27(23.7%)

In the Kokompe community, the study found out that the greater majority of the participants sampled were in the 62–95 year range with a frequency of 13 (representing 37.14% of the sampled population). The 18–28 year category recorded only one (1) person representing about 2.86% of the sampled population (Table 1).

In the Tanfiano community, the study showed that the frequency of participants in the various age categories were similar (Table 1). The age categories 29–39, 40–50 and 62–95 all had frequencies of 8 each representing 21.05% of the total patients sampled in the community.

#### Parasitological indices of onchocerciasis

The parasitological indices of onchocerciasis within the study population (*n* = 114) is summarized in Table 2. It is noteworthy that every community in this study had MDA ongoing for more than 10 years. For the entire study population, there were 7 people (6%) who were carriers of microfilaria. Tanfiano had the highest prevalence of microfilaria carriers (13.2%; *n* = 5), followed by Kokompe (2.9%; n = 1) and Senya (2.43%; n = 1), but the difference was not statistically significant (*p* = 0.2717, *one-way ANOVA*).

**Table 2:** The Prevalence of Nodules and Microfilaria (mf) of the Study Population

Study Community	Rounds of ivermectin	No. of cases examined	Prevalence of nodule careers N; (%)	Prevalence of Mf carriers N;(%)
Tanfiano	20	38	2 (5.3%)	5 (13.2%)
Senya	20	41	2 (4.9%)	1 (2.4%)
Kokompe	18	35	5 (14.3%)	1(2.9%)
Total	–	114	9 (7.89%)	7(6.14%)

The association between nodule prevalence and microfilaria prevalence was variable. The Tanfiano community recorded the highest microfilaria prevalence among the study communities. The microfilaria prevalence in Tanfiano was 13.2%, which was more than 2 times higher than the prevalence of onchocercal nodules (5.3%), though the difference was not statistically significant (*p* = 0.8553, *Welch’s unequal variances t-test*). In Senya, the prevalence of onchocercal nodules was 4.9%, about 2 times higher than the microfilaria prevalence which was 2.4%, however, the difference was not statistically significant (*p* = 0.2546, *Welch’s unequal variances t-test*). In Kokompe however, the prevalence of nodules (14.3%) was about 5 times higher than the microfilaria prevalence (2.9%) albeit the difference was just not statistically significant (*p = 0.0580*, *Welch’s unequal variances t-test*).

#### Gender distribution of nodules and microfilaria in the study communities

The prevalence of nodules and microfilaridermia among females and males in the study communities is presented in Table 3. Females in the study communities had a higher prevalence of microfilaria carriers (8.6%) relative to that of the males (5.4%), though the difference was not statistically significant (*p = 0.7526, Chi Square test*). The prevalence of nodules was slightly higher in females (8.6%) compared to males (7.1%), however, again the difference was not statistically significant (*p = 0.7699*, *Chi Square test*).

**Table 3:** Gender Distribution of Nodules and Microfilaria Prevalence in the Study Communities

Gender	No. of cases examined	Prevalence of Mf Carriers N; (%)	Prevalence of nodule carriers (%)
Females	58	5 (8.6%)	5 (8.6%)
Males	56	3 (5.4%)	4 (7.1%)
Total	114	8 (7%)	9 (7.9%)

### Clinical manifestations of onchocerciasis in the study communities

The summary of findings on onchocerciasis-associated clinical manifestations are presented in Table 4. The most frequent clinical manifestation observed in this study was dermatitis. This was followed by ocular onchocerciasis (visual impairment and blindness). The percentage of individuals with dermal and ocular onchocerciasis in the 3 study areas were 33.3% (38/114) and 12.3% (14/114) respectively.

**Table 4:** Clinical Manifestations of Onchocerciasis in the Three Study Communities of the Nkoranza North District of Ghana

Study Community	No. of cases examined	No. of people with Nodules (N; %)	Dermatitis (N;%)	Visual impairment (N;%)	Blindness (N;%)
Tanfiano	38	2 (5.3%)	6 (15.8%)	4 (10.5%)	0 (0.0%)
Senya	41	2 (4.9%)	12 (29.3%)	3 (7.3%)	2 (4.9%)
Kokompe	35	5 (14.3)	11 (31.4%)	2 (5.7%)	3 (8.5%)
Total	114	9 (7.89%)	29 (25.44%)	9(7.89%)	5(4.4%)

The relationship between dermatitis and microfilaredermia is presented in Table 5. A large number of non- microfilaridermic people also had dermatitis as 25 people who were microfilaria negative reported that they experienced dermatitis. The number of people who were microfilaria positive and had episodes of dermatitis was almost the same as those who were microfilaria positive but did not experience any dermatitis.

**Table 5:** The Relationship between Dermatitis and Microfilaridermia among the Study Participants

Data analyzed	MF positive	MF Negative	Total
Dermatitis	4	25	29
No Dermatitis	3	82	85
Total	7	107	114

## Discussion

This study is the first published work on the prevalence of onchocerciasis and its associated clinical manifestations in the Nkoranza North District of the Bono East Region. Thus, the findings are important to help direct the onchocerciasis control programme of the district as well as nearby districts with similar characteristics.

The study demonstrated that the microfilaria level for all 3 communities was below 20%. This means that the study areas are hypoendemic for onchocerciasis based on the criteria used by the Onchocerciasis Elimination Programme for the Americas (OEPA), where nodule and microfilarial prevalence of ≤20%, 21–59% and ≥ 60% are defined as hypoendemic, mesoendermic and hyperendemic respectively [27]. This finding is interesting because the district of the study area was previously classified as mesoendemic for onchocerciasis by the Ghana Health Service [28]. Consequently, the present observations suggest a general downward change in prevalence of the disease. This supports the frequently reported effectiveness of ivermectin as a microfilaricide. However, the community of Tanfiano which had received over 20 rounds of ivermectin had a higher (though statistically not significant) microfilaria prevalence than the other two communities. This finding could be due to the fact that the community frequently received quite a number of migrants from areas such as Kintampo, Atebubu, Prang and other surrounding communities [29] which are known to be hyper or mesoendemic for onchocerciasis.

In this study, the prevalence of both microfilaria and nodules were higher in females than in males. Gender related nodule prevalence has been reported in other studies and it has been attributed to the differences in degree and frequency of duration of how males and females participants are exposed to the bites of infective *Simulium* vectors, with female’s participants being more exposed [30, 31]. Their increased exposure can be due to certain roles and activities such as washing by river banks and fetching water for drinking and domestic purposes [30, 31]. However, some other studies have found contrasting results as the prevalence of onchocerciasis in males in those areas were higher than the prevalence in females. The differential exposure to bites of blackfly fly vectors has been shown to be dependent on the behaviour, occupation and clothing of different gender which vary from one culture to the next [32].

The study also investigated the prevalence of certain clinical manifestations which are often associated with river blindness. It was found that the most frequently encountered clinical symptom in the study communities was dermatitis, followed by visual impairment, nodules and blindness. Visual damage is considered to be the most serious clinical feature of onchocerciasis, which may affect all tissues of the eye [4]. Ocular lesions are usually seen only in those with moderate or heavy microfilarial loads [33]. Although onchocerciasis is commonly known as river blindness, the ocular manifestations of the disease actually only occur in about 5% of those affected [4]. However for this study, the prevalence of ocular manifestations is 12% which is relatively higher than the normal prevalence of ocular onchocerciasis frequently reported [4], though other authors have reported higher estimates in other countries [34].

Another interesting finding from this study was the fact that a large number of amicrofilaridermic people also had dermatitis. This might be possible because ivermectin is a known effective microfilaricide, as it is able to clear skin microfilaria and in some places has been proven to halt transmission of onchocerciasis [15, 18, 19]. Thus, people infected with onchocerciasis who take ivermectin during MDAs might have the microfilaria cleared from their skin, though some of the chronic clinical manifestations of onchocerciasis might persist. However, despite the strong microfilaricidal and embryostatic effects of ivermectin, several investigators have demonstrated that it has negligible or no macrofilaricidal effects [35–37]. This has necessitated the search for new or repurposed antifilarial drugs which have adulticidal effects on *O. volvulus* worms [38]. In this respect, the use of doxycycline which has been proven to have adulticidal effects on *Wolbachia* can be considered as doxycycline results in amelioration of clinical manifestations of the disease [38]. There is however the need for more research in this regard as the usage of doxycycline in MDA programmes has some logistical challenges [38]. Some of these challenges include contraindications in children below 8 and pregnant women as well as concerns that if doxycycline is used on a large scale such as in MDA programmes, resistance to the antibiotic might develop [39]. Meanwhile, doxycycline remains one of the major “weapons” for the treatment of a variety of atypical infections [39, 40].

## Conclusions

The results from this study demonstrated that onchocerciasis remains a health problem in the study area despite several years of ivermectin administration. Though the drug seems to be effective in reducing microfilaria prevalence in the communities, the persistence of the disease provides some cause for concern. Constant surveillance of the disease is thus warranted in order to help review the frequency of MDA with ivermectin in the district. There is also an increasing urgency for research and development towards new antifilarial drugs which have macrofilaricidal effects to complement ivermectin.

## Abbreviations

DALYs: Disability adjusted life years
MDA: Mass drug administration
OEPA: Onchocerciasis Elimination Programme for the Americas
SOR: Sub-optimal response
WHO: World Health Organization
